# Editorial Comment: Feasibility and safety of irreversible electroporation (IRE) in patients with small renal masses: Results of a prospective study

**DOI:** 10.1590/S1677-5538.IBJU.2020.03.11

**Published:** 2020-02-20

**Authors:** M Buijs, PJ Zondervan, DM de Bruin, KP van Lienden, A Bex, OM van Delden, Stênio de C. Zequi

**Affiliations:** 1 University of Amsterdam Academic Medical Center Department of Urology Amsterdam Netherlands Department of Urology, Academic Medical Center, University of Amsterdam, Amsterdam, the Netherlands; 2 University of Amsterdam Academic Medical Center Department of Urology Amsterdam Netherlands Department of Urology, Academic Medical Center, University of Amsterdam, Amsterdam, the Netherlands; 3 Academic Medical Center Department of Biomedical Engineering and Physics Amsterdam Netherlands Department of Biomedical Engineering and Physics, Academic Medical Center, Amsterdam, the Netherlands; 4 Academic Medical Center Department of Radiology Amsterdam Netherlands Department of Radiology, Academic Medical Center, Amsterdam, the Netherlands; 5 Antoni van Leeuwenhoek Hospital The Netherlands Cancer Institute Department of Urology Amsterdam Netherlands Department of Urology, The Netherlands Cancer Institute, Antoni van Leeuwenhoek Hospital, Amsterdam, the Netherlands; 1 Fundação Prudente A. C. Camargo Cancer Center São PauloSP Brasil A. C. Camargo Cancer Center, Fundação Prudente, São Paulo, SP, Brasil

## COMMENT

The options for therapy of patients presenting with small renal masses (SRM) is growing. Besides the partial nephrectomy or active surveillance, the percutaneous ablation performed through radiofrequency (FRFA) or cryoablation (CRYO) has been used in the last two decades. Beyond RFA and CRYO, new alternatives for ablation of kidney tumors has been proposed:

The percutaneous microwave ablation (PMWA), that is is no time-consuming procedure being also used also in complex cystic masses. Shapiro et al compared the use PMWA in 40 patients with T1b non metastatic renal cell carcinoma versus partial nephrectomy or radical nephrectomy. At end of study, complications were low, and functional and oncologic results were similar to surgical cases (the majority of the series). Patients undergone PMWA was older and presented more comorbidities. PMWA seems to be one more option for sick or old patients presenting tumors in pT1b stage, that are not included in traditional definition of SMR (< 4.0 cm).

Another recent option is irreversible electroporation, that has the first results published from a prospective Phase 2 Dutch trial, from Buijs et al. The treatment is based in repetitive electric pulses generated between needles (electrodes); that promotes destabilization on cellular membranes of target lesion, resulting in cell death without promoting thermal damage of adjacent tissues. The procedure was safe, and painless, however required a long surgical anesthesia time (usually IRE is a quick procedure), requiring a progressive skill of the teams evolved.

Both articles were on small casuistries, suggesting that these initial results are competitive with actual therapies. New technologies for ablation of renal tumors are welcome, in face the progressive increase of the diagnostic of SRM that has been concomitant with the widespread global population aging, that a risk factor for this malignance.

